# Uncoupling of ATP-Mediated Calcium Signaling and Dysregulated Interleukin-6 Secretion in Dendritic Cells by Nanomolar Thimerosal

**DOI:** 10.1289/ehp.8881

**Published:** 2006-03-21

**Authors:** Samuel R. Goth, Ruth A. Chu, Jeffrey P. Gregg, Gennady Cherednichenko, Isaac N. Pessah

**Affiliations:** 1 National Institute of Environmental Health Sciences Center for Children’s Environmental Health; 2 Department of Veterinary Molecular Biosciences and; 3 Department of Medical Pathology, University of California–Davis, Davis, California, USA; 4 MIND (Medical Investigation of Neurodevelopmental Disorders) Institute, University of California–Davis, Sacramento, California, USA

**Keywords:** calcium, calcium channel, dendritic cell, ethyl mercury, immunotoxicity, interleukin-6, organic mercury, redox, thimerosal

## Abstract

Dendritic cells (DCs), a rare cell type widely distributed in the soma, are potent antigen-presenting cells that initiate primary immune responses. DCs rely on intracellular redox state and calcium (Ca^2+^) signals for proper development and function, but the relationship between these two signaling systems is unclear. Thimerosal (THI) is a mercurial used to preserve vaccines and consumer products, and is used experimentally to induce Ca^2+^ release from microsomal stores. We tested adenosine triphosphate (ATP)-mediated Ca^2+^ responses of DCs transiently exposed to nanomolar THI. Transcriptional and immunocytochemical analyses show that murine myeloid immature DCs (IDCs) and mature DCs (MDCs) express inositol 1,4,5-trisphosphate receptor (IP_3_R) and ryanodine receptor (RyR) Ca^2+^ channels, known targets of THI. IDCs express the RyR1 isoform in a punctate distribution that is densest near plasma membranes and within dendritic processes, whereas IP_3_Rs are more generally distributed. RyR1 positively and negatively regulates purinergic signaling because ryanodine (Ry) blockade *a*) recruited 80% more ATP responders, *b*) shortened ATP-mediated Ca^2+^ transients > 2-fold, and *c*) produced a delayed and persistent rise (≥ 2-fold) in baseline Ca^2+^. THI (100 nM, 5 min) recruited more ATP responders, shortened the ATP-mediated Ca^2+^ transient (≥ 1.4-fold), and produced a delayed rise (≥ 3-fold) in the Ca^2+^ baseline, mimicking Ry. THI and Ry, in combination, produced additive effects leading to uncoupling of IP_3_R and RyR1 signals. THI altered ATP-mediated interleukin-6 secretion, initially enhancing the rate of cytokine secretion but suppressing cytokine secretion overall in DCs. DCs are exquisitely sensitive to THI, with one mechanism involving the uncoupling of positive and negative regulation of Ca^2+^ signals contributed by RyR1.

Recent animal and human studies have underscored the strong influence of genetic, epigenetic, and physiologic factors in defining susceptibility of the immune system to methylmercury (MeHg) and ethylmercury (EtHg) (Havarinasab and Hultman 2005; Lawler et al. 2004; Silbergeld et al. 2005). Immune dysregulation triggered by organic mercury can include suppression, stimulation, loss of tolerance, and generation of auto-antibodies. Therefore, the pattern of immunotoxicity induced by organic mercury is likely to depend not only on the chemical form, timing, and dose to which an individual is exposed but also on susceptibility factors that are poorly understood at present. Thus, significant attention is currently focused on identifying which types of immune cells and biomolecules are critical targets of low-level organic mercury and their functional consequences on overall immune status.

Sodium ethylmercurithiosalicylate (thimerosal; THI) is an EtHg-containing compound used to preserve cosmetics, blood products, and vaccines and is also used experimentally to induce calcium (Ca^2+^) release from microsomal [endoplasmic reticulum/sarcoplasmic reticulum (ER/SR)] stores in intact cells. THI toxicity is due to the EtHg moiety. THI and EtHg toxicity in humans consist of a few cases of accidental high-dose poisoning (Cinca et al. 1980; Damluji 1962; Zhang 1984). Attention has been focused on THI in vaccines, where it is used as a preservative for multiuse formulations. THI was withdrawn from pediatric vaccines starting in 1999 (Centers for Disease Control and Prevention 1999) over concerns that organic mercury is a known neurodevelopmental toxicant. Nevertheless, THI is still used in influenza, diphtheria toxoid, diphtheria toxoid and acellular pertussis (DTaP), and tetanus toxoid vaccines. The hypothesis that THI can cause neurodevelopmental disorders was tested by injecting THI and THI-containing vaccines into inbred strains of young mice (Hornig et al. 2004). Growth, behavioral, and histologic abnormalities in the brains of the autoimmune susceptible strain (SJL) were recorded after administration of THI or THI plus vaccine. Autoimmune-resistant strains (C57BL/6, BALB/c) did not display any of the abnormalities, suggesting a strong influence of inherent immune status and the neurodevelopmental toxicity of THI.

We hypothesized that especially sensitive targets of THI-mediated immune dysregulation are dendritic cells (DCs), whose function is to acquire antigens derived from self or nonself sources and efficiently present them to naive and resting T cells (Banchereau and Steinman 1998). This hypothesis stems from the fact that ambient oxygen (O_2_) tension or thiol concentration directly influences DC secretion of interferon-γ (IFN-γ) and interleukin-12 (IL-12) (Murata et al. 2002), enhances expression of FcɛR1, the high affinity receptor for IgE (Novak et al. 2002), and regulates surface class II major histo-compatibility complex (MHC) expression (Goth et al. 2006) *in vitro*. In this regard, Ca^2+^ contributes essential signals for DC function and maturation. Differentiation (Bagley et al. 2004), pro-inflammatory cytokine secretion (Gardella et al. 2000), apoptotic cell phagocytosis (Poggi et al. 1998), and migrational responsiveness to purine nucleotides or chemokines (Partida-Sanchez et al. 2004; Scandella et al. 2004) are Ca^2 +^-dependent processes. DCs rely on changes in intracellular redox state and Ca^2+^ signals for proper development and function, but the relationship between these signaling systems in DCs is unclear.

THI contains an oxidized mercury atom (Hg^2+^) whose redox properties can enhance the activity of the inositol 1,4,5-trisphosphate receptor (IP_3_R) and ryanodine receptor (RyRs), both intracellular Ca^2+^ channels (Kaplin et al. 1994; Pessah et al. 2002). THI elicits Ca^2+^ release from ER/SR stores in lymphocytes (Bultynck et al. 2004) and ER/SR microsomes by targeting the IP_3_R and RyR (Abramson et al. 1995; Kaplin et al. 1994). THI-treated, monocyte-derived DCs failed to or only minimally phosphorylated STAT (signal transducer and activator of transcription) proteins 1, 3, 4, and 6, implying that the JAK (janus kinase) signaling pathway and, by extension, cytokine receptors are bypassed in the sensitization phase induced by THI (Valk et al. 2002). DCs express several classes of Ca^2+^ channel proteins that mediate Ca^2+^ signals. DCs express store-operated Ca^2+^ channels (Hsu et al. 2001) and IP_3_Rs that regulate release of Ca^2+^ from ER/SR stores in response to adenosine triphosphate (ATP) (Schnurr et al. 2003) and chemokines (Scandella et al. 2004). Immature DCs (IDCs) express message for one of three genetic forms of the RyR, the RyR1 (Hsu et al. 2001; O’Connell et al. 2002). The influence of THI and its metabolites EtHg and thiosalicylic acid (TSA) on Ca^2+^ signaling and activation of DCs remain unexplored.

To study how THI and EtHg influence Ca^2+^-dependent DC functions, we generated and tested murine DCs under normoxia (5% O_2_ vol/vol) and omitted 2-mercaptoethanol (2-Me) from the culture medium. In this article we report that DCs primarily express the type 1 isoform of the IP_3_R and RyR ER/SR Ca^2+^ channels, known targets of THI. THI and ryanodine (Ry) each block early positive contributions of the RyR1 to ATP-induced Ca^2+^ transients and uncouple inhibitory feedback, indicating a common mechanism. The consequences of THI upon ATP-induced IL-6 production, a Ca^2+^-dependent process, were examined. THI initially enhanced the IL-6 secretion rate, but ultimately suppressed its accumulation. DCs are exquisitely sensitive to THI, with one prominent mechanism involving the uncoupling of positive and negative regulation of Ca^2+^ signals contributed by RyR1.

## Materials and Methods

### Chemicals and antibodies

THI (USP grade) and its metabolite TSA, propidium iodide (PI), diethylpyrocarbonate, and Na_2_ATP were purchased from Sigma (St. Louis, MO). We purchased fibronectin (bovine plasma) from Calbiochem (San Diego, CA) and ethyl-mercuric chloride (EtHgCl) from ICN (Costa Mesa, CA). Recombinant murine granulocyte/macrophage colony stimulating factor (GM-CSF) was purchased from Sigma or R&D Systems (Minneapolis, MN), as were murine IL-6 ELISA kits. Antibodies (BD-Pharmingen, San Diego, CA) are as follows (clone name): class II MHC-biotin (2G9), CD11c-APC (HL3), CD16/32 (2.4G2), and hamster immunoglobulin. Anti-RyR monoclonal antibody 34C (recognizes types 1 and 3) was purchased from the Developmental Studies Hybridoma Bank (Iowa City, IA). Anti-IP_3_R and anti-IP_3_R1 polyclonal antibodies were purchased from Chemicon (Temecula, CA). Prolong Antifade, Fura-2 AM, Fluo-4 AM, Alexa 488–conjugated goat anti-mouse IgG antibody, Alexa 488–conjugated goat anti-rabbit IgG antibody, and Alexa 647–conjugated goat anti-rabbit IgG antibody were purchased from Invitrogen (Carlsbad, CA). 7-Aminoactinomycin D (7AAD) was purchased from Calbiochem. A fluorescent terminal deoxynucleotidyl transferase (TdT) labeling kit was purchased from Promega (Madison, WI).

### Cell culture

Female C57BL/6J mice 6–8 weeks of age were purchased from JAX West, Inc. (Davis, CA), treated humanely and with regard for alleviation of suffering, and euthanized in accordance with a protocol approved by the University of California–Davis Animal Resources Service. Bone-marrow–derived DCs were generated by modifying a protocol (Lutz et al. 1999), using normoxia (5% O_2_ vol/vol), and omitting 50 μM 2-Me from the culture medium (Goth et al. 2006). R10 medium was RPMI 1640 (Invitrogen) with 10% fetal bovine serum (FBS; Hyclone, Logan, UT), 2 mM l-glutamine, 2 mM sodium pyruvate, 100 IU/mL penicillin, and 10 μg/mL streptomycin. Cultures were maintained at 37°C in a Thermo Forma model 3130 incubator (Thermo Forma, Marietta, OH) equipped with a CO_2_ and fuel-cell O_2_ monitor and N_2_ and CO_2_ gas supplies. CO_2_ was set to 5% vol/vol, and O_2_ to 5% vol/vol, and were periodically verified using Fyrite gas analyzers (Bacharach Inc., New Kensington, PA).

### DC cytometric flow sorting and analysis

Cells were flow sorted between culture days 6 and 10. A detailed description of our cell preparation for flow cytometry has been published (Goth et al. 2006). Briefly, nonadherent cells were preblocked with 2.4G2 monoclonal antibody (1.0 μg/mL) and hamster IgG (0.25 μg/mL) for 10 min. Fluorescent anti-class II MHC (0.1 μg/mL) and anti-CD11c (0.3 μg/mL) monoclonal antibodies were added and allowed to bind for 15 min. After washing with 2% FBS in phosphate-buffered saline (PBS), cells were aseptically sorted on a MoFlo cytometer (Cytometric, Fort Collins, CO). Single-stained and unstained controls were used to define sorting gates and to adjust compensation. CD11c-positive cells were considered DCs and graded as IDCs or mature DCs (MDCs) depending on their class II MHC expression. We routinely obtained ≥ 85% purities of sorted IDC and MDC subsets. PI was added to a final concentration of 0.5 μg/mL before sorting or before analysis on a FACScan flow cytometric analyzer (Becton Dickinson, Palo Alto, CA) to detect dead cells.

### DC treatment

THI, EtHgCl, and TSA solutions were dissolved in sodium carbonate using borosilicate glass pipettes and tubes. Dilutions were made in R10 and used within 1 hr. DCs (1–2 × 10^6^ cells/mL) were aliquoted into perfluorocarbon tissue culture vials or 96-well format plates (Savillex, Minnetonka, MN). R10 or medium containing THI, EtHgCl, TSA, or lipopolysaccharide (LPS) (to 1 μg/mL) was added, and cells were placed in a 37°C incubator.

### DC transcriptome analysis

Total RNA was isolated from sorted DCs using Trizol Reagent (Molecular Research Center, Cincinnati, OH) according to the manufacturer’s recommended procedure. DCs were resuspended to 1 × 106 cell/mL in R10 media, plated in per-fluorocarbon containers, and incubated for 20 hr. Biotinylated cRNA was synthesized from 5 μg of total cellular RNA according to the protocol published by Affymetrix Inc. (Affymetrix 2004). Fragmented, labeled cRNA was hybridized onto Affymetrix mouse 430A or 430 2.0 GeneChip arrays. Microarrays were hybridized 16 hr at 45°C, stained, and washed according to an Affymetrix protocol (EukGE-WS2v4; Affymetrix 2004). Fluorescence intensity was measured with a scanner equipped with Affymetrix Microarray Analysis Suite version 5.0. The average intensity for each array was normalized by scaling to a target intensity value of 125, allowing comparison between arrays. Individual transcripts are represented by perfect-match probes in conjunction with a corresponding set of mismatch probes. A transcript is called present if the average intensity value of perfect-match cells is ≥ 1.5 times greater than the average intensity of mismatch cells, and the average intensity difference between perfect-match and mismatch cells is four or more times the experimental noise. Poorly performing probes (where the ratio between the average intensity of mismatch cells and perfect-match cells is four or more times the experimental noise) were not included in the analysis. RNA from three independent cultures was analyzed on GeneChips (i.e., three GeneChips per treatment were analyzed).

### Immunocytofluorescence of calcium channels

DCs were washed in 1% bovine serum albumin (BSA) in PBS, centrifuged onto glass slides using a Cytofuge2 (Statspin, Norwood, MA), air dried, fixed with 4% paraformaldehyde in PBS for 20 min at 4°C, and then permeabilized with three washes of 0.2% Tween-20 in PBS (TPBS). Nonspecific binding was blocked with goat IgG (50 μg/mL). Cells were incubated with primary antibody (dilutions were 1:20 34C and 1:100 anti-IP_3_R and IP_3_R1 polyclonal antibodies in TPBS) for 1 hr. Blocking peptide for the IP_3_R1 antibody was used at the manufacturer’s suggested concentration. After washing, Alexa 488– or Alexa 647–conjugated anti-mouse and anti-rabbit secondary antibodies were diluted 1:1,000 in TPBS and allowed to bind 1 hr. Cells were washed with TPBS and then with PBS. After mounting with Prolong Antifade plus 40 μg/mL 7AAD, DCs were visualized for immunofluorescence using an MRC 600 laser scanning confocal microscope (Bio-Rad, Richmond, CA). Confocal immunopheno-typing was performed on two separate cultures.

### TdT assay

IDCs were treated with 500 nM TSA, THI, or medium for 20 hr as described above. Cells were then washed twice with ice-cold PBS and fixed with 4% paraformaldehyde in PBS for 20 min on ice. Cells were washed in 1% BSA in PBS and resuspended to 0.5 × 106 cells/mL in 1% BSA in PBS; cell aliquots were then spun onto microscope slides. Slides were air dried at least 2 hr and then immersed in 4% diethylpyrocarbonate:ethanol prechilled to –20°C for 30 min to stop endogenous nuclease activity. After washing twice with PBS, cells were processed for DNA strand end-labeling according to the protocol supplied by the kit manufacturer (Promega). After TdT labeling, nuclei were counterstained with 1 μg/mL PI in water for 15 min before mounting in Prolong Antifade for confocal imaging.

### [^3^H]Ryanodine binding analysis

High-affinity binding of [^3^H]ryanodine ([^3^H]Ry; 56 or 50 Ci/mmol; PerkinElmer, Boston, MA) to rabbit skeletal microsomes enriched in RyR1 was performed as previously described (Pessah et al. 1987). Nonspecific binding was determined by including 1,000-fold unlabeled Ry. Data were reported in picomoles of bound Ry per milligram of protein.

### IL-6 assays

IDCs were pulsed with 100 nM THI or TSA for 20 min, pelleted, supernatant aspirated, and resuspended to 2 × 10^7^/mL; 0.05 mL of the cell suspension was aliquoted per well into a perfluorocarbon 96-well plate, and 0.05 mL ATP (0, 0.2, 2, or 20 μM final) was added per well. Medium and LPS (1 μg/mL final) cells received no pre-treatment. Supernatants for IL-6 ELISA were collected 20 hr later from the top portion of the cultures. IL-6 concentrations were interpolated from the linear response range of the cytokine standard; minimum sensitivity was 7 pg/mL.

### Calcium imaging

IDCs were resuspended in R10 supplemented with 5 ng/mL recombinant murine GM-CSF to 0.5 × 10^6^/mL; 0.5 mL was plated overnight onto fibronectin-coated glass coverslips. The next day, cells were labeled with 5 μM Fura-2 AM or Fluo-4 AM for 20 min. Cells were washed with bathing solution (130 mM NaCl, 4 mM KCl, 10 mM HEPES, 10 mM glucose, 2 mM CaCl_2_, 2 mM MgSO_4_, pH 7.3, with NaOH) and imaged within 1 hr. Changes in cytoplasmic Ca^2+^ were measured by emission at 510 nm (Fluo-4) or ratioing emission at 510 nm with excitation pair 340/380 nm (Fura-2) (Fessenden et al. 2003). Rapid perfusion of ATP and caffeine was accomplished by a micropipette above the cells being imaged (Automate, Oakland, CA).

### Data analysis

Nonlinear curve fitting analysis and one-way analysis of variance were performed using Origin 6.0 (OriginLab Corporation, Northampton, MA) software to test for statistical significance.

## Results

Culturing murine bone marrow at physiologic O_2_ tension (5% vol/vol) without 2-Me supplementation generated myeloid IDCs and MDCs with a similar yield, leukocyte marker expression, and allostimulatory capacity as DCs produced from cultures using ambient (20%) O_2_ and 50 μM 2-Me (Goth et al. 2006). A representative flow cytometric dot plot showing sorting gates for IDCs and MDCs is shown in Supplemental Material, Figure 1A (available online at http://www.ehponline.org/docs/2006/8881/suppl.pdf). RNAs extracted from sorted IDCs and MDCs were analyzed using gene probe arrays. IDCs and MDCs have a common lineage, and a small number of changes in gene expression occurred during maturation. Of this limited set of genes, class II MHC mRNA expression is down-regulated with maturation, whereas CD86 message was up-regulated with maturation (Table 1). Glyceraldehyde-3-phosphate dehydrogenase (GADPH) was expressed at similar levels in IDCs and MDCs (Table 1). Both DC subsets express two Ca^2+^ channel types that are targets of THI. Of the three IP_3_R isoforms, IDCs and MDCs express message for ITPR1 and ITPR3, the latter down-regulated with maturation (Table 1). Of the three RyR isoforms, RyR1 mRNA is present in both DC subsets, RyR3 is expressed upon maturation, and RyR2 is not expressed (Table 1). Because the Ca^2+^ channel genes detected encode targets of THI, we explored the distribution of the proteins in IDCs using confocal microscopy.

**Figure 1:**
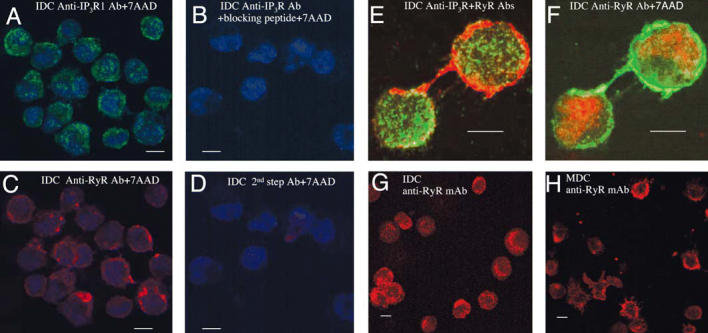
DCs express IP_3_R and RyR Ca^2+^ channels. Abbreviations: Ab, antibody; mAb, monoclonal antibody. (*A*–D) IDCs labeled with anti-IP_3_R1 (*A*) or anti-RyR (*C*) or with anti-IP_3_R1 plus blocking peptide (*B*) or fluorescent second-step Ab alone (*D*). Nuclei were counterstained with 7AAD; magnification, 100×. (*E* and *F*) Merged images of RyR1 and IP_3_R (pan anti-IP_3_R) immunostaining in IDCs (*E*), and RyR1 and 7AAD nuclear staining (*F*); images were acquired at 100× with 2.5× digital magnification. (*G* and *H*) IDCs (*G*) and MDCs (*H*) stained for RyR; magnification, 40×. Bars = 10 μm.

IDCs express IP_3_R1 in a dense granular distribution in a pattern consistent with targeting to ER membranes (Figure 1A,E). In contrast, intense RyR1 staining localizes near the plasma membrane, and foci of protein extend from the base into dendrites (Figure 1C,E,F). IDCs lack detectable RyR1 within perinuclear regions (Figure 1C,E,F). Cells stained with secondary antibody alone or with antibody-blocking peptide (IP_3_R1) had no detectable signal (Figure 1B,D). The codistribution of RyR1 and IP_3_R was further examined using an anti-RyR1 monoclonal antibody and a pan anti-IP_3_R polyclonal antibody. The RyR1 is densely localized within a narrow band at the cell periphery and extends within dendrites (Figure 1E,F), whereas IP_3_R extends to regions lacking RyR1 (Figure 1E). The granular appearance and distribution of IP_3_R protein in IDCs did not change when visualized with an isoform specific (IP_3_R1) or pan-IP_3_R antibody (Figure 1A,E). The distribution of RyR proteins in IDCs and MDCs (using a monoclonal antibody specific for RyR1 and 3) were similar (Figure 1G,H) despite the additional expression of RyR3 transcripts in MDC (Table 1).

**Figure 2:**
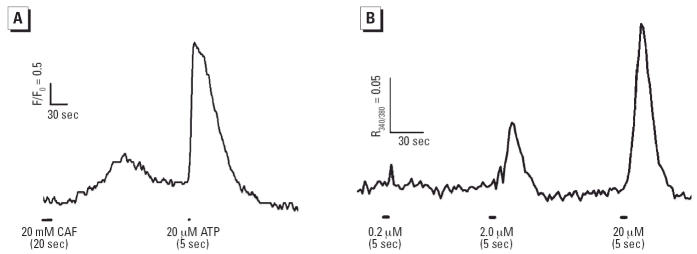
IDCs respond to caffeine (CAF) and ATP. (*A*) IDCs were loaded with Fluo-4 or Fura-2 as described in “Materials and Methods.” IDCs that responded to CAF (20 mM) also responded to 20 μM ATP, and cells that failed to respond to ATP also did not respond to caffeine (data not shown). (*B*) The ATP response magnitude (averaged trace of 20 cells shown) was dose dependent. (*A*) and (*B*) represent two independent experiments.

Ca^2+^ responses of IDCs to the RyR agonist caffeine were tested. Given the expression of RyR1 within IDCs, and that MDCs respond to caffeine (Schnurr et al. 2003), we expected IDCs would respond to caffeine with a rise in cytoplasmic Ca^2+^. Approximately 50% of IDCs responded to 20 mM caffeine (Figure 2A). Because extracellular ATP induces maturity in IDCs (la Sala et al. 2001, 2002; Schnurr et al. 2001) and intracellular Ca^2+^ contributes an essential DC maturation signal (Bagley et al. 2004), we decided to test the ability of our IDCs to respond to extra-cellular ATP. IDCs responded vigorously to a brief (5 sec) application of ATP, the response amplitude dose-dependent between 0.2 and 20 μM (Figure 2B). Therefore, ATP potently elicits Ca^2 +^ transients, likely mediated through agonist actions on P2Y purinergic nucleotide receptors expressed in IDCs (Idzko et al. 2002). We next generated viability–dose survival curves for DCs exposed to THI and its metabolites EtHg (from EtHgCl) and TSA [chemical structures are shown in Supplemental Material, Figure 1B (available online at http://www.ehponline.org/docs/2006/8881/suppl.pdf)].

**Figure 3:**
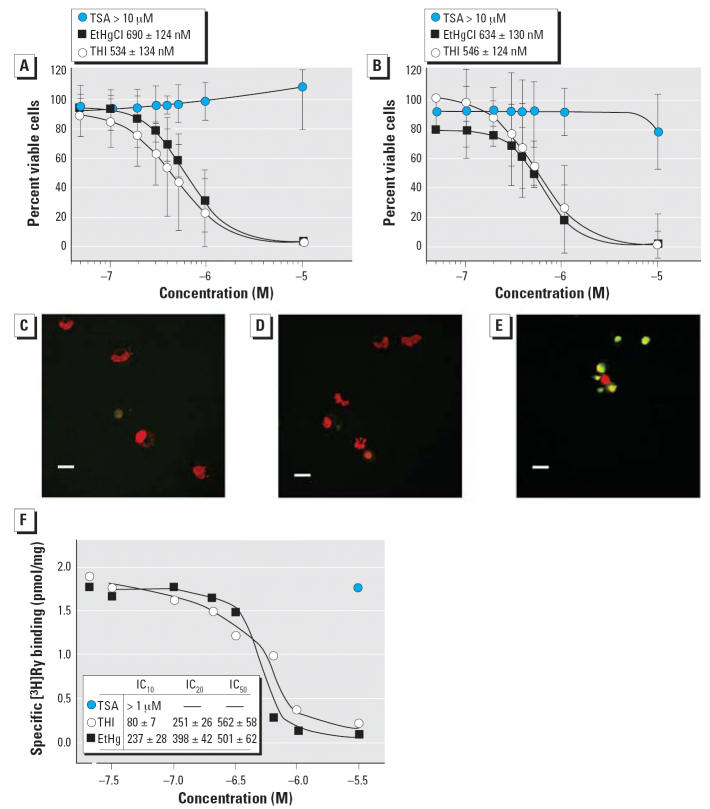
Dose–response survival curves and IC_50_ values for IDCs and MDCs treated 20 hr with THI, EtHgCl, or TSA. (*A* and *B*) Survival curves plotting percent PI-negative cells versus medium control (as 100%) for IDCs (*A*) and MDCs (*B*). (*C–E*) TdT DNA strand labeling and confocal microscopy of IDCs treated with medium (*C*), 500 nM TSA (*D*), or 500 nM THI (*E*) for 20 hr. Nuclei with strand breakage stain green; all nuclei are counterstained red with PI. Note the small and round apoptotic morphology of the THI-treated nuclei compared with controls. Original magnification, 40×; bars = 10 μm. (*F*) THI and EtHgCl inhibit [^3^H]Ry binding to RyR1 high-affinity sites. Receptor binding analysis was performed as described in “Materials and Methods”; IC_10_, IC_20_, and IC_50_ values were determined by nonlinear curve fitting. TSA had no effect at the highest concentration tested. Data shown are the average of two independent experiments.

Range-finding experiments determined that a 20 hr exposure to 10 μM THI consistently killed > 90% of DCs using flow cytometry, judged by their increased permeability to PI and decreased cell size [forward light scatter (FSC); data not shown]. THI, EtHgCl, and TSA were titrated from 50 nM to 10 μM, IDC and MDC subsets were treated for 20 hr, and cell PI permeability was measured by flow cytometry. Figure 3A and B shows that THI and EtHgCl caused dose-dependent decreases in DC viability compared with the TSA control. The viability dose–response curves for THI and EtHgCl were the same within each DC subset and were similar between the cell subsets. IC_50_ (concentration that inhibits 50%) values for the DC subsets treated with THI, TSA, and EtHgCl are shown in Figure 3A and B. DC death triggered by THI could be mediated by apoptosis or primary necrosis, because the FSC and PI uptake data acquired 20 hr posttreatment cannot distinguish between the two possibilities. IDCs were treated with medium alone, 500 nM TSA, or 500 nM THI for 20 hr and probed for DNA strand breakage, an indicator of apoptosis (Figure 3C,D,E, respectively). Only THI-treated cells demonstrate both increased 2′-deoxyuridine 5′-triphosphate-fluorescein isothiocyanate labeling and a corresponding shrinkage in nuclear size (Figure 3E); death from THI exposure is therefore likely to be an apoptotic outcome. The action of THI and EtHgCl upon RyR1 channels was further explored by measuring [^3^H]Ry binding to ER/SR membranes enriched in RyR1. THI and EtHgCl inhibited the specific binding of [^3^H]Ry to RyR1 (Figure 3F; IC_50_ = 562 and 501 nM).

**Figure 4:**
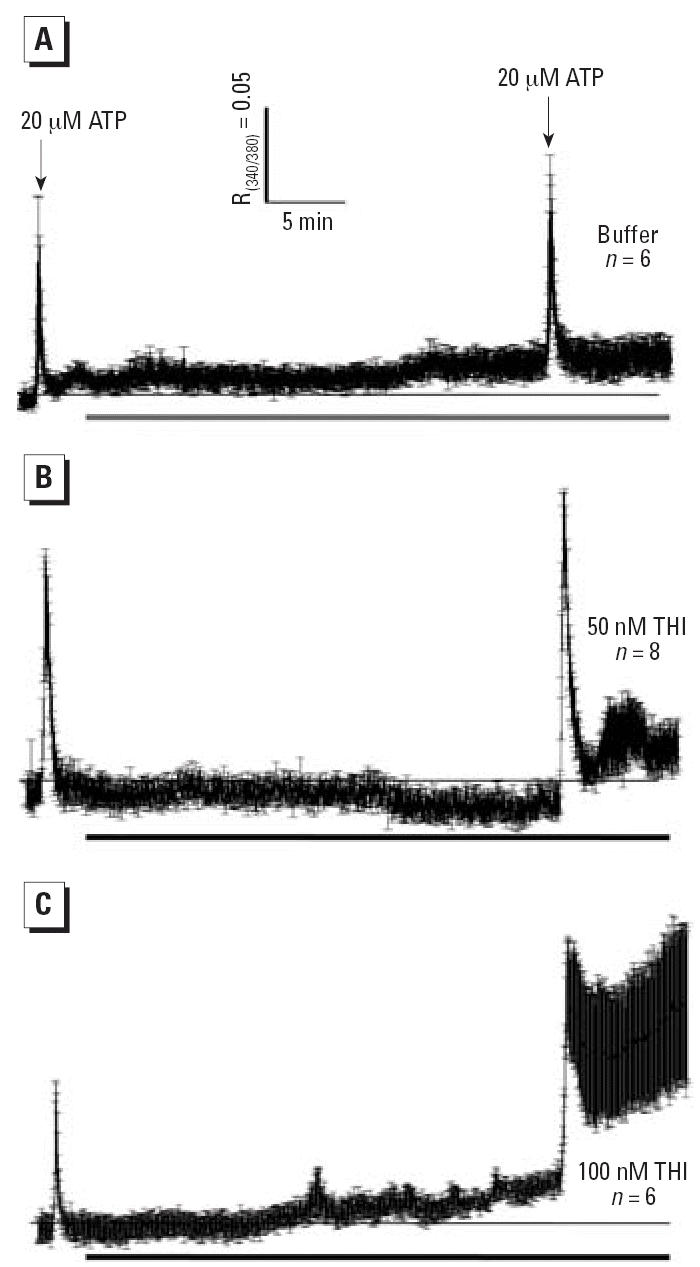
Ca^2+^ transients elicited in IDCs by ATP before and after 20 min exposure (bars beneath traces) to buffer (*A*), 50 nM THI (*B*), or 100 nM THI (*C*). DCs were given 20 μM ATP (5 sec, first arrow) 1 min after the start of trace recording. The cells were challenged a second time with ATP, and traces were recorded 8 min. Thin horizontal lines indicate the initial baseline. R_340/380_ ratiometric data were acquired every 2 sec. Traces are the mean ± SE and represent two experiments.

Lymphocyte tolerance or immunity to an antigen can be driven by IDCs or MDCs, respectively (Steinman et al. 2005), and we focused on characterizing the effects of THI upon ATP-mediated Ca^2+^ signaling (a maturation signal) in IDCs. THI can release Ca^2+^ from ER/SR stores by selectively enhancing the activity of IP_3_R and RyR, and we examined its actions on ATP-mediated Ca^2+^ signaling in IDC. ATP is an efficacious activator of DCs through its agonist actions on P2Y purinergic nucleotide receptors (Idzko et al. 2002). P2Y receptors couple to Gαq protein that initiates signal transduction events leading to the hydrolysis of phosphatidylinositol 4,5-bisphosphate (PIP_2_) to IP_3_ and diacylglycerol. IP_3_ in turn activates IP_3_R that mobilizes Ca^2+^ from ER stores (Di Virgilio et al. 2001a, 2001b). IDCs challenged with two pulses of 20 μM ATP (5 sec each) 30 min apart (with constant perfusion) produced Ca^2+^ transients whose peak heights, baseline to peak rates, or peak to baseline decays were not significantly different (Figure 4A). This indicates that the concentration, duration, and frequency of the ATP challenges did not desensitize the cell’s capacity to respond, nor were they additive.

Perfusion of 50 nM THI after an initial ATP test pulse (Figure 4B) did not induce a rise in baseline Ca^2+^ in the 30 min of perfusion that preceded the second ATP challenge. In the presence of THI, the second ATP pulse produced a baseline to Ca^2+^ peak height comparable with that elicited by the first ATP challenge. However, the rate of decay of the response peak was significantly slowed by THI compared with control cells. THI also induced a sustained elevation in the 8 min of trace recording in intracellular Ca^2+^ after ATP withdrawal. DCs exposed to 100 nM THI (Figure 4C) showed a gradual rise in resting Ca^2+^. Although cells responded to a second ATP challenge, decay of the response was significantly slower and incomplete compared with DCs exposed to 50 nM THI. After withdrawal of ATP from the perfusion medium, a more pronounced slow rise of intracellular Ca^2+^ concentration was seen and remained elevated for the duration of the measurement. Some DCs tested with 20 μM ATP failed to show detectable responses. However these “ATP-resistant” cells invariably responded vigorously to ATP after exposure to 50 nM THI for 30 min (data not shown). We next dissected the ATP-mediated calcium wave in DCs using pharmacologic agents known to modify components of Ca^2+^ signaling.

**Figure 5:**
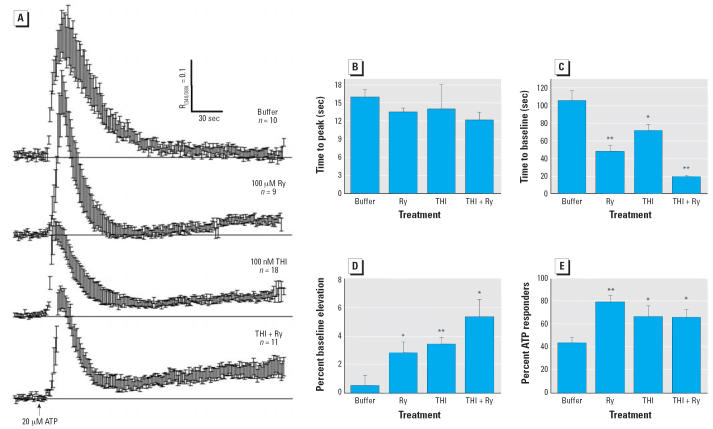
Ry and THI functionally uncouple IP_3_R1 and RyR1 in IDCs. (*A*) Ca^2+^ transients elicited by ATP (20 μM, 5 sec) applied to IDCs that were preincubated either with buffer (top trace), 100 μM Ry for 4 hr (second trace), 100 nM THI (5 min; third trace), or 100 μM Ry (4 hr) + 100 nM THI (5 min) (bottom trace). Traces are mean responses ± SE and represent one of two experiments. (*B*–*E*) Statistics (mean ± SE) for time to peak (*B*), time to baseline (*C*), percent baseline elevation (*D*), and percent ATP responders (*E*) derived from the cells shown in (*A*). Neither Ry nor THI alone or combined alter time to peak response toward 20 μM ATP (*B*). However, Ry and/or THI significantly shortened the recovery time to baseline (*C*), persistently elevated the recovery baseline for resting [Ca^2+^] (*D*), and recruited more ATP-responsive DCs (*E*). Results were replicated in three independent experiments. *p < 0.05. **p < 0.01.

ATP generated a stereotyped Ca^2+^ transient having a time to peak of 16.1 ± 2.6 sec and a decay time of 106 ± 2.6 sec (Figure 5A, top trace). To test the RyR1’s contribution to ATP-mediated Ca^2+^ transients, IDCs were pre-treated 4 hr with 100 μM Ry, which irreversibly locks the RyR in a nonconducting conformation (Buck et al. 1992; Zimanyi et al. 1992). ATP-challenged Ry-treated cells produced transients whose time to peak did not significantly differ from control (Figure 5A, second trace; Figure 5B). However, after ATP withdrawal, the recovery time to baseline was > 2-fold (*p* < 0.01) faster (Figure 5A, second trace; Figure 5C) and showed a delayed and persistent rise (*p* = 0.036) in baseline Ca^2+^ after triggering (Figure 5A, second trace; Figure 5D). A 5 min THI (100 nM) pre-exposure mimicked the effects of Ry. When challenged with ATP, the rise time remained unchanged (Figure 5B), but the Ca^2+^ transient was shortened > 1.5-fold (*p* = 0.05; Figure 5C) and the delayed rise in Ca^2+^ baseline was prominent (*p* = 0.02) (Figure 5A, third trace; Figure 5D).

The effects of THI and Ry in combination were nearly additive (Figure 5A, fourth trace; Figure 5C,D), suggesting a common mechanism targeting RyR1 function. THI and Ry alone or combined increased the number of IDCs responding to ATP 1.5- to 1.8-fold compared with control (*p* < 0.05; Figure 5E). THI’s actions on IDCs Ca^2+^ signaling were not seen with TSA; therefore, they were mediated by organic mercury (data not shown). These results (Figures 4, 5) indicate that as little as 50–100 nM THI in a short time span (5–30 min) potentiates agonist-mediated Ca^2+^ signaling events in DCs that primarily manifest as a prolonged elevation in intracellular Ca^2+^. DCs are arguably the most sensitive target cell for THI identified to date, and this sensitivity to oxidative insult may reflect a unique manner in which they generate and use Ca^2+^ signals in response to a changing redox environment.

**Figure 6:**
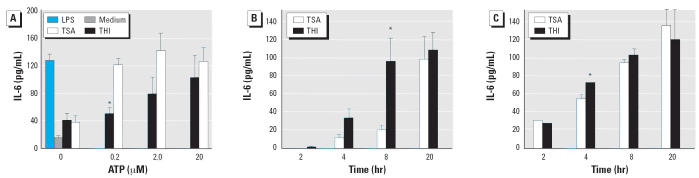
THI suppression or exacerbation of ATP-mediated IL-6 secretion from IDCs is dependent on the ATP dose. (*A*) IL-6 accumulation induced by 0.2 μM ATP is suppressed by 100 nM THI. Kinetics of IL-6 secretion induced by 2 μM ATP (*B*) or 20 μM ATP (*C*) is enhanced by 100 nM THI. DCs were pretreated with THI or TSA as in (*A*). Data are means of quadruplicates ± SE. An independent cell culture was used for each graph. *p > 0.05.

Increased intracellular Ca^2+^ induced by inhibitors of the ER/SR Ca^2+^-ATPase is associated with the rapid secretion of macrophage IL-6 (Bost and Mason 1995). DCs produce IL-6 in response to IL-1β, tumor necrosis factor-α, or LPS, and other myeloid cells secrete IL-6 in response to ATP (Shigemoto-Mogami et al. 2001). We hypothesized that THI-induced uncoupling of ATP-mediated Ca^2+^ signaling would disrupt IL-6 secretion. IDCs were pretreated with 100 nM THI or TSA and challenged with graded concentrations of ATP, and secreted IL-6 was measured. Figure 6A shows that IDCs pretreated with THI or TSA alone secreted low levels of IL-6 that did not significantly differ from the medium control. LPS, which induces IL-6 synthesis by non-Ca^2+^-dependent pathways, induced a large increase in IL-6. All three ATP concentrations induced comparable amounts of IL-6 by the TSA-pretreated DCs. By 20 hr, these IL-6 concentrations were equal to the LPS-treated control. Pretreating IDCs with THI and challenging with ATP attenuated IL-6 secretion compared with TSA-pretreated controls, reaching significance at the lowest (0.2 μM) ATP dose. This lowered IL-6 secretion was not due to cell death because this was overcome by 2 and 20 μM ATP. Next, we determined the kinetics of ATP-mediated IL-6 secretion in THI-treated DCs to see if cytokine production was attenuated at earlier time points.

Figure 6B shows that 2 μM ATP induced IL-6 by 4 hr, consistent with its rapid induction in other myeloid cells. THI pretreatment accelerated IL-6 secretion compared with TSA controls, indicating that THI sensitized DCs to ATP. Maximal IL-6 was secreted by THI-treated DCs by 8 hr, whereas controls needed an additional 12 hr. A concentration of 20 μM ATP was more potent than a 2 μM concentration, eliciting IL-6 by 2 hr and near-maximal levels at 8 hr in both THI- and TSA-pretreated control IDCs (Figure 6C). The strong accelerating effect of THI versus TSA on IL-6 secretion induced by 2 μM ATP was not as pronounced with 20 μM ATP, generating a small but significant increase 4 hr after its application (Figure 6C).

## Discussion

DC activation and associated immune functions are subject to regulation by their redox environment (Bagley et al. 2004; Gardella et al. 2000; Goth et al. 2006; Murata et al. 2002; Novak et al. 2002). We generated DCs under tightly regulated O_2_ and without 2-Me to provide a more physiologic baseline to study the mechanism of redox active environmental triggers such as THI and EtHg in regulating DC activation *in vitro*. For DCs, Ca^2+^ signaling events provide an essential “upstream” component, engaging immediate events such as cytokine production and secretion (Ferrari et al. 2000; la Sala et al. 2002) and long-term (e.g., maturational) responses. Importantly, microsomal IP_3_R and RyR Ca^2+^ channel functions are tightly regulated by changes in local redox state (Bultynck et al. 2004; Kaplin et al. 1994; Pessah et al. 2002).

We show for the first time the expression and distribution of two major Ca^2+^ channel proteins expressed in DCs. We have provided transcriptomal and direct immunocytochemical evidence that DCs express specific isoforms of the IP_3_R and RyR Ca2+ channels. RyR and IP_3_R proteins distinctly distribute in DC subsets. In IDCs, RyR1 and IP_3_R1 localize below the plasma membrane at the base of the dendrites and into the processes. A similar distribution of the RyR was seen in MDCs. However IP_3_Rs extend to reticular regions where the RyR1 is either absent or at very low levels. In human monocytes, IP_3_R and RyR localization patterns similar to our murine DCs were found (Clark and Petty 2005).

**Figure 7:**
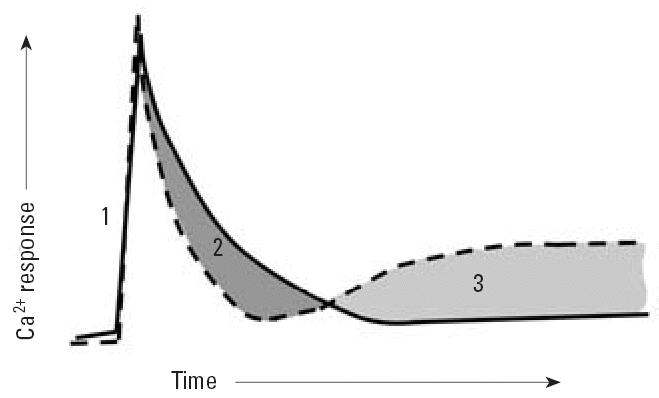
Model showing how THI and Ry uncouple regulation of Ca^2+^ transients elicited by ATP in IDCs. The solid line depicts a typical Ca^2+^ transient triggered by ATP in naive IDCs (Figure 4A). Under these conditions, IP_3_R1 and RyR1 are functionally coupled. The dashed line indicates observed changes in the ATP-triggered Ca^2+^ transient after pre-incubation with THI (100 nM, 5 min) or RyR1 channel block (100 μM Ry, 4 hr). The rising phase of the transient (phase 1) remains unchanged by either THI or Ry treatments (alone or combined) and is generated by IP_3_R activation. Transient termination is significantly faster after RyR1 modification by THI or Ry (alone or combined; phase 2). Modified RyR1, IP_3_R1, and/or Ca^2+^ entry may become “leaky,” resulting in a persistent elevation in resting Ca^2+^ (phase 3). Modification of RyR1 conformation by either THI or Ry is sufficient to uncouple phases 2 and 3 (see “Discussion” for details).

ATP-triggered Ca^2+^ transients in IDCs have three components that engage cross-talk between IP_3_Rs and RyRs. The initial rate and amplitude of the Ca^2+^ transient (phase 1; Figure 7) is largely dependent upon IP_3_R activation. It is unlikely the RyR1 has a significant influence on phase 1 because blocking RyR1 channels with Ry has no measurable consequence on these parameters. The transient decay rate depends on both IP_3_R and RyR1 because blocking RyR1 channels with Ry significantly enhances this rate back to baseline (phase 2; Figure 7). Ca^2+^-induced Ca^2+^ release, presumably mediated by activation of RyR1, must therefore contribute to slowing the decay rate.

However, RyR1 activity in IDCs also contributes to the negative regulation, reestablishing a stable resting Ca^2+^ level near that before activation of purinergic signaling (phase 3; Figure 7). Phase 3 was unmasked when Ry or THI modified RyR1 conformation. Phases 2 and 3 are coupled events that rely on the conformational state of the RyR1. In this regard, both micromolar Ry and nanomolar THI uncouple functional cross-talk between IP_3_R and RyR1 channels normally regulating purinergic signaling in IDCs.

Collectively, these data show that RyR1 channels are closely coupled to IP_3_-induced Ca^2+^ release and contribute to the temporal properties of the transient by prolonging the decay and restoring the original resting Ca^2+^ level. RyR1 therefore contributes both positive and negative regulation to purinergic signaling in IDCs. THI, like Ry, appears to uncouple RyR1 functions from phosphoinositide signaling in IDCs in a concentration- and time-dependent manner.

Nanomolar THI deregulated ATP-mediated signaling by a mechanism that uncoupled phases 2 and 3 of the Ca^2+^ transient. THI did not appear to influence the initial response to ATP by activation of the IP_3_R (phase 1) but enhanced the transient decay rate after agonist withdrawal (phase 2) and elicited a persistent rise in intracellular Ca^2+^ (phase 3). THI’s actions on IP_3_R-mediated signals have not been previously explored in DCs. However, evidence indicates that IP_3_R1 is a target of THI. Using triple-IP_3_R-knockout R23-11 cells derived from DT40 chicken B lymphoma cells, THI (1–100 nM) potentiated IP_3_-induced Ca2+ release when IP_3_R1, but not IP_3_R3, is expressed (Bultynck et al. 2004).

RyR1 is also a sensitive target of THI (Figure 3E) (Abramson et al. 1995). We show that ATP triggers a Ca^2+^ transient in IDCs whose temporal property relies on functional coupling of IP_3_R and RyR1 channels that is extremely sensitive to THI (≤ 100 nM). How THI sensitizes RyR1 to activation by Ca^2+^ involves release of the inhibitory actions of Mg^2+^, an important physiologic modulator (Donoso et al. 2000; Sanchez et al. 2003) and may be mediated by hyperreactive cysteines within the RyR1 channel complex (Liu and Pessah 1994; Voss et al. 2004). Exactly how THI alters ATP-dependent and -independent Ca^2+^ signals may involve multiple molecular mechanisms involving the IP_3_R1, RyR1, and possibly other channels. Nevertheless, the present results reveal that IDCs are a particularly sensitive target of THI, with as little as 50 nM within 30 min uncoupling ATP-induced Ca^2+^ transients.

DC sensitivity to their oxidative environment may reflect how they generate and use Ca^2+^ signals in response to a changing redox environment. Redox modulation of DC function is underscored by our finding that THI modulates IL-6 synthesis elicited by exogenous ATP. Myeloid DC IL-6 strongly influences mucosal T-cell and gut B-cell responses. Lung DCs with intrinsic T_H_2-polarizing activities generated T_H_1 responses from naive CD4+ T cells in the presence of anti–IL-6 neutralizing antibody (Dodge et al. 2003). Peyer’s patch B cells were induced to secrete IgA by IL-6 elaborated by local CD11b-positive DCs; IgA induction was reduced by anti–IL-6 antibodies (Sato et al. 2003).

*In vivo* ATP steady-state levels are at nanomolar and low micromolar (1–25 μM) concentrations in bulk fluids and at the cell surface, respectively (Lazarowski et al. 2003). At these concentrations, metabotropic G-protein–coupled P2Y receptors are engaged. In THI-treated DCs, IL-6 production kinetics are enhanced by 2 μM and 20 μM ATP, and IL-6 secretion is significantly suppressed by 0.2 μM ATP (Figure 6A). THI-enhanced IL-6 secretion may be a consequence of prolonged calcium signals resulting from the uncoupling effects of THI toward IP_3_R- and RyR1-mediated signals. Increased intracellu-lar Ca^2+^ is associated with IL-6 RNA stabilization and rapid IL-6 secretion (Bost and Mason 1995). THI-mediated attenuation of IL-6 secretion at 0.2 μM ATP was not unexpected because the suboptimal calcium signals induced by 0.2 μM ATP may not have been sufficient to promote maximal rates of IL-6 synthesis. Ca^2+^-stimulated RyR1 channels are initially activated and then inactivated by THI (Figure 3F) (Abramson et al. 1995). Over the 20 hr assay used in this study, the uncoupling of IP_3_R–RyR1 functions is likely to have broad effects upon Ca^2+^-dependent processes.

A practical implication of the present findings has relevance to the commercial uses of THI as an antimicrobial agent in vaccines and consumer products because they identify DCs as sensitive targets for THI- and EtHg-mediated dysfunction. Given the importance of DCs as a front line in regulating lymphocyte-mediated immunity and tolerance, altering DC functions by forms of EtHg should be considered when assessing contributions to altered immune function. In studies using the autoimmune-susceptible A.SW (H-2^s^) mouse strain, THI induces a syndrome that is stronger and more generally manifested than those produced by methylmercury (Havarinasab et al. 2004), and development of autoimmunity in H-2^s^ mice is dependent on cellular (T cell) and soluble (IFN-γ and IL-6) factors (Havarinasab et al. 2005). Onset of spontaneous systemic autoimmune disease symptoms (proteinuria, anti-DNA antibodies) in (NZBxNZW)F_1_ mice is hastened by THI (Havarinasab and Hultman 2006). Interestingly, disease morbidity and mortality in these F_1_ mice are dramatically reduced by neutralizing anti–IL-6 receptor antibody (Mihara et al. 1998), an effect associated with reduced IgG (auto-) antibody production.

The human *RyR1* gene is highly polymorphic. More than 60 single missense or deletional mutations have been closely linked to the pharmacogenetic disorders malignant hyperthermia and central core disease (Gronert et al. 2004). Our findings that DCs primarily express the RyR1 channel complex and that this complex is uncoupled by very low levels of THI with dysregulated IL-6 secretion raise intriguing questions about a molecular basis for immune dyregulation and the possible role of the RyR1 complex in genetic susceptibility of the immune system to mercury.

## Figures and Tables

**Table 1:** Mean signal intensities of Ca^2+^ channel and selected immune marker gene expression in bone-marrow–derived IDCs and MDCs from C57BL/6 mice.

	IDCs		MDCs	
Gene product (GenBank accession no.)	Signal^a^	Call	Signal	Call
RyR1 (X83932.1)	99, 360, 178	P	59, 114, 107	P
RyR2 (NM_023868.1)	8, 2, 2	A	18, 6, 2	A
RyR3 (AV238793)	13, 3, 13	A	34, 75, 48	P
IP_3_R1 (NM_010585.1)	77, 148, 147	P	183, 178, 181	P
IP_3_R2 (NM_019923.1)	31, 23, 32	A	50, 27, 46	A
IP_3_R3 (NM_080553.1)	116, 128, 115	P	87, 95, 68	P
MHC class II H2-IAβ (M15848.1)	2,493, 4,467, 3,727	P	1,454, 2,340, 1,775	P
CD86 (NM_019388.1)	167, 399, 596	P	398, 721, 669	P
GADPH (NM_008084.1)	11,241, 4,761, 4,547	P	11,126, 4,635, 5,186	P

Abbreviations: A, transcript absent; P, transcript present. DCs were cultured without 2-Me at 5% O_2_; data are from three independent DC cultures. GenBank data are available online (GenBank 2006).

a Relative RNA expression; individual values are given for three assays: the first value is from hybridization to an Affymetrix mouse 430A GeneChip array, and the second and third values are from hybridization to an Affymetrix 430 2.0 GeneChip array.
