# Supplementary material for: Uncoupling of ATP-Mediated Calcium Signaling and Dysregulated Interleukin-6 Secretion in Dendritic Cells by Nanomolar Thimerosal
Source: Environ Health Perspect. 2006 Mar 21;114(7):1083–91. doi: 10.1289/ehp.8881 (PMC1513334; doi:10.1289/ehp.8881)
Supplement: Supplemental Figures and Tables [file ehp0114-001083s1.pdf]

Supplementary Figure 1 Goth, et al.

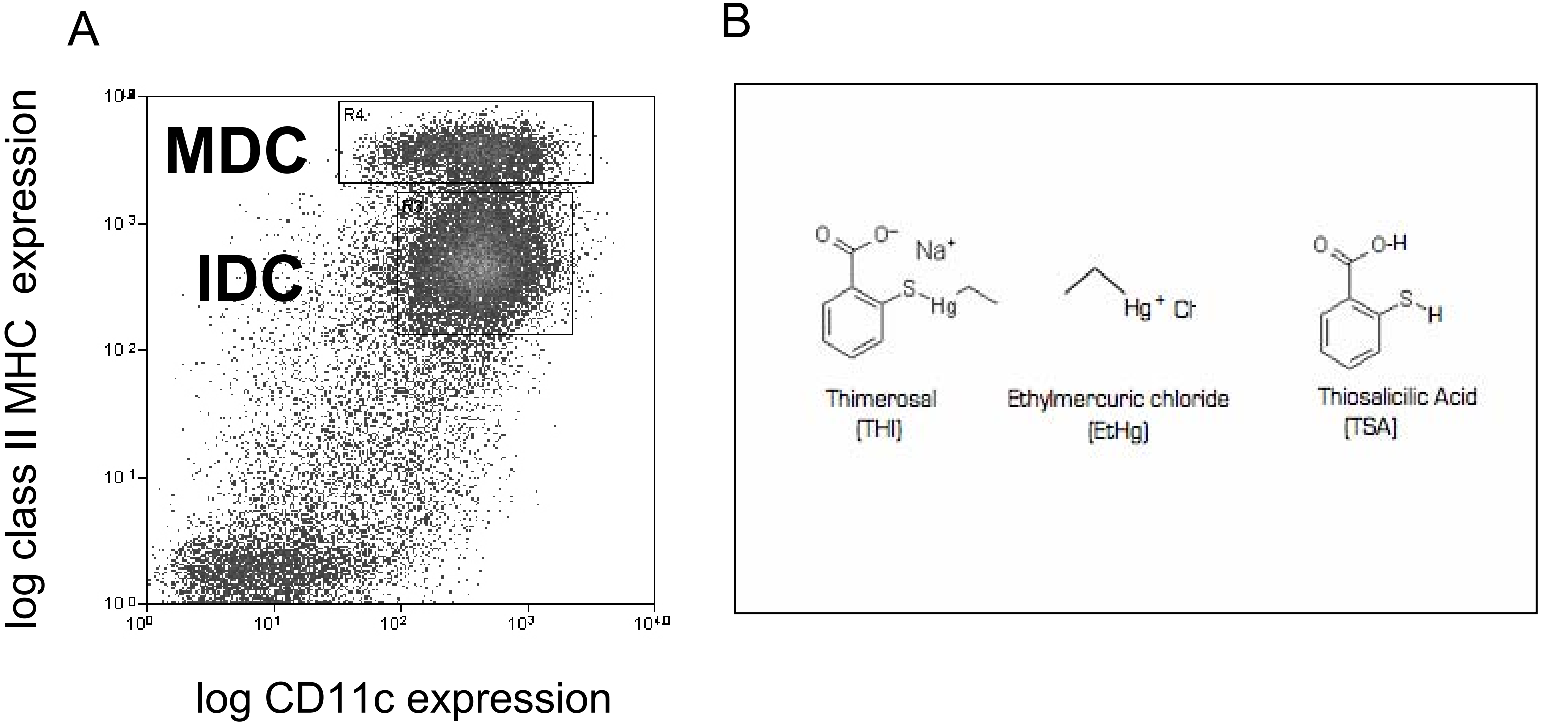

A. Representative flow cytometric dot plot showing CD11c vs. class II MHC expression in the bone marrow derived dendritic cell (DC) cultures. used in this study. Thin line boxes within the dot plot are the gates used to select mature and immature DCs (MDC, IDC) for cell sorting.

B. Line structure drawings of the compounds thimerosal, ethylmercuric chloride and thiosalicylic acid used in this study. Ethylmercuric chloride and TSA ionize in solution to form the THI metabolites ethylmercuric and thiosalicylate ions.
